# Investigation of Ser315 Substitutions within *katG* Gene in Isoniazid-Resistant Clinical Isolates of *Mycobacterium tuberculosis* from South India

**DOI:** 10.1155/2015/257983

**Published:** 2015-01-28

**Authors:** A. Nusrath Unissa, N. Selvakumar, Sujatha Narayanan, C. Suganthi, L. E. Hanna

**Affiliations:** Division of Biomedical Informatics, Department of Clinical Research, National Institute for Research in Tuberculosis (NIRT), Indian Council of Medical Research (ICMR), No. 1 Mayor Sathyamoorthy Road, Chetput, Chennai, Tamil Nadu 600 031, India

## Abstract

Mutation at codon 315 of *katG* gene is the major cause for isoniazid (INH) resistance in *Mycobacterium tuberculosis* (*M. tuberculosis*). Substitution at codon 315 of *katG* gene was analyzed in 85 phenotypically resistant isolates collected from various parts of southern India by direct sequencing method. The obtained results were interpreted in the context of minimum inhibitory concentration (MIC) of INH. Of the 85 phenotypically resistant isolates, 56 (66%) were also correlated by the presence of resistance mutations in the *katG* gene; 47 of these isolates had ACC, 6 had AAC, 2 had ATC, and one had CGC codon. The frequency of Ser315 substitution in *katG* gene was found to be higher (70%) amongst multidrug-resistant (MDR) strains than among non-MDR (61%) INH-resistant isolates. Further, the frequency of mutations was found to be greater (74%) in isolates with higher MIC values in contrast to those isolates with low MIC values (58%). Therefore, the study identified high prevalence of Ser315Thr substitution in *katG* gene of INH-resistant isolates from south India. Also, isolates harboring this substitution were found to be associated with multidrug and high level INH resistance.

## 1. Introduction

Although tuberculosis (TB) is a preventable, treatable, and curable disease, it still remains as a major public health problem. The period of 6–9 months needed to treat the disease is too long, and the treatment is often associated with significant toxicity. These factors make patient compliance to therapy very difficult, leading to the emergence and selection of drug-resistant TB bacteria [1]. The emergence of multidrug-resistant (MDR) TB, defined as strains which are resistant to two most potent anti-TB drugs, namely, isoniazid (INH) and rifampicin (RIF), and XDR-TB, defined as MDR-TB strains that are resistant to second-line TB drugs, that is, fluoroquinolones and at least one of the injectable aminoglycosides (capreomycin, kanamycin) or amikacin, has worsened the situation further [2]. Globally, 450,000 people developed MDR-TB in 2012; more than half of these cases were from India, China, and the Russian Federation. It is estimated that about 9.6% of MDR-TB cases had XDR-TB. About 170,000 MDR-TB deaths are estimated to have occurred in 2012 [3].

Resistance against all known anti-TB drugs has been reported. However, isolates of* M. tuberculosis* resistant to INH are seen with increasing frequency (1 in 10^6^) as compared to isolates resistant to other drugs [4]. Also, resistance to INH, alone or in combination with other drugs, is now the second most common cause for resistance. Globally, it has been estimated that INH resistance was found in 10.3% of new cases and 27.7% of treated cases [5]. An earlier study based on susceptibility testing from south India also reported high resistance to INH in 15.4% of cases compared to 4.4% cases of RIF resistance [6].

INH has been used extensively as the frontline anti-TB drug and a drug of choice for chemoprophylaxis, acting as a principle component in the current six-month short course chemotherapy regimen. It has long been recognized that INH resistance in* M. tuberculosis* correlates with the loss of catalase and peroxidase (CP) activity in resistant strains [7–9]. In other words, INH resistance is often accompanied by loss and/or reduction of CP or KatG activity coded by* katG* gene. In fact, INH is a prodrug that requires cellular activation by KatG protein to its active form, before it can exert its toxic effect on the bacillus [7]. There has been considerable interest to understand the molecular basis of INH resistance. Mutations in several genes (*katG*,* inhA, ahpC, kasA*, and others) have been associated with INH resistance [10–13]. Of these,* inhA* and* ahpC* promoter mutations lead to low-level INH resistance, whereas mutation in* katG* is responsible for high-level resistance. Zhang et al. (1992) demonstrated that mutation in the* katG *gene, coding for KatG protein, is a major contributor to INH resistance in* M. tuberculosis *[10].

The most prevalent mutation found to occur in* katG *gene is AGC to ACC at codon 315 [14], or Ser315Thr (S315T) substitution. The appearance of this mutation was most frequent amongst MDR strains [15]. However, this mutation was also reported to be associated with intermediate or high levels of resistance to INH (1 to 10 *μ*g/mL) [16]. When compared to other resistance-conferring mutations in* katG*, S315T substitution was found to result in near-normal CP activities. Also it maintains the levels of virulence and confers resistance to INH simultaneously [17]. Therefore, it may be considered as a reliable biomarker for the detection of INH resistance.

There are very few studies [18–20] on molecular characterization of INH resistance from India and there is paucity of data for south Indian isolates. Previous studies have clearly shown that the south Indian INH-resistant (INH^R^) isolates were quite distinct and had lower virulence and higher susceptibility to H_2_O_2_ [21–23]_._ Hence, it was of interest to analyze mutations in* katG *gene encompassing codon 315 in INH^R^ clinical isolates of* M. tuberculosis* from south India by DNA sequencing method in the present study.

## 2. Methods

### 2.1. INH-Resistant Clinical Isolates of* M. tuberculosis*

A total of 85 INH^R^ clinical isolates of* M. tuberculosis* were randomly selected from south Indian TB patients (20–60 years old) belonging to both the sexes from 2006 to 2009 at the National Institute for Research in Tuberculosis (NIRT), Chennai, India. Of the 85 isolates collected, 67 were from Tamil Nadu, 14 were from Andhra Pradesh, 3 were from Karnataka, and one was from Kerala. Drug susceptibility testing (DST) was performed on the cultures; they were coded and subjected to DNA sequencing for identification of mutations in the* katG* gene.

### 2.2. DNA Extraction from INH-Resistant Strains

Genomic DNA was prepared from the resistant strains using sodium chloride and cetyltrimethylammonium bromide method as described previously [18].

### 2.3. Amplification of* katG* Gene

Amplification was performed in the isolated genomic DNA using a reaction mixture containing 1 *μ*L of forward and reverse primers (10 pmol) each, 6 *μ*L of deoxyribonucleoside triphosphates (dNTPs) mix (2.5 mM), 2.5 *μ*L of 10X PCR buffer, 10–50 ng of template genomic DNA, and 1 U of* Taq* DNA polymerase (Amersham Biosciences, UK). The amplification was performed in a thermal controller (MJ Research, USA) with 30 cycles (1 minute (min) at 95°C, 30 seconds at 63°C, and 1 min at 72°C, followed by a final extension step at 72°C for 10 min). The primers of* katG *gene forward sequence (5′-A A A C A G C G G C G C T G G A T C G T 3′) and reverse sequence (5′-G T T G T C C C A T T T C G T C G G G G 3′) were used to generate a 209 bp fragment encompassing the S315T codon [24]. The amplicons were purified using GFX COLUMN (Amersham Biosciences, UK) according to the manufacturer's instructions.

### 2.4. DNA Sequencing

Sequencing of the amplicon was carried out using an automated DNA sequencer (ABI Prism 310 Genetic Analyzer-Applied Biosystems, USA), using the above-mentioned primers and the BigDye terminator sequencing kit (Applied Biosystems). To 4 *μ*L of the terminator ready reaction mix, l *μ*L of the amplified fragment (2-3 ng) and 1 *μ*L of the primer (1 pmol/*μ*L) were added and the volume was made up to 20 *μ*L using deionised water. The reaction mixture was subjected to cycle sequencing. The samples were vortexed and spun, then heated at 95°C for 2 min, and immediately chilled on ice. They were vortexed and spun again and placed on ice and loaded onto the DNA sequencer. We refer to supporting documents for the figures (see Supplementary Material available online at http://dx.doi.org/10.1155/2014/257983). The sequence obtained was compared with sequences available in EMBOSS database via http://www.ebi.ac.uk/emboss/align/ using the alignment tool. The GenBank accession number for* katG* is X68081.

## 3. Results and Discussion

On the basis of DST results, 46 strains were found to have a minimum inhibitory concentration (MIC) of 1 mg/liter (L), 17 had an MIC of 5 mg/L, and the remaining strains had MICs of >5 mg/L. Amongst the 85 isolates, 51% were MDR cases, and 48% were found to be INH monoresistant cases with or without resistance to other TB drugs (data not shown). A laboratory reference strain of* M. tuberculosis*, H_37_Rv, was used as the control. Of the 85 phenotypically INH^R^ isolates included in the present study, 56 isolates (66%) were found to possess resistance conferring mutations to INH in the* katG *gene upon genotyping. The remaining 29 phenotypically resistant isolates had no known mutations in the* katG *gene. Of the 56 isolates with mutations, 47 had ACC (Thr), 6 had AAC (Asn), two had ATC (Ile), and one had CGC (Arg) substitution at codon 315 (Table 1). This observation suggests a high frequency distribution of S315 substitutions in south Indian isolates, which is consistent with the global pattern [14]; therefore, this region may be regarded as a hot spot region. The predominance of 315 mutations in the* katG* gene in INH^R^ clinical isolates has been documented in several studies, with observed frequencies ranging between 30% and >90%. In countries such as Scotland, Spain, Italy, and Uruguay [25–28] the prevalence of INH^R^ strains with S315T substitution was found to be relatively low (20–50%). Studies from countries like Netherlands, India, Africa, China, and Dubai have reported moderate levels of prevalence of the 315 substitutions, ranging between 50 and 70% [16, 18, 19, 29–31]. The S315T substitutions have been reported to be highly prevalent (60 to >90%) among INH^R^ strains circulating in counties like South Africa, Russia, Brazil, Lithuania, and Peru [24, 32–35].

In this study, the frequency of S315T substitution in* katG* gene was slightly higher (70%) in MDR strains than in non-MDR (61%) INH^R^ isolates. It has also been previously observed that S315T substitution is associated with MDR strains of* M*.* tuberculosis*. Further, the mutation has important implications for the transmission and control of MDR-TB [17]. We also observed a strong correlation between the MICs of INH and occurrence of S315T substitutions in our isolates with 74% of them showing high MIC values (≥5 mg/L) and were associated with S315T substitution, while only 58% of isolates with low MIC value of 1 mg/L had this mutation. These findings are in agreement with the observations made earlier by van Soolingen et al. [16].

In one our previous study, we compared virulence using five clinical mutants of KatG and found that the virulence of S315 mutants from south India was comparable to that of wild type H_37_Rv [36]. This is in contrast to the classical notion that INH^R^ *M. tuberculosis *isolates from south India are inherently less virulent than fully susceptible organisms [22, 23]. Our present findings on the high prevalence of S315 mutants in south Indian isolates suggest their wider transmission. Moreover, Cohen et al. (2004) provided ecological evidence to support the theory that mutations at position 315 of* katG *confer INH resistance in* M. tuberculosis *without diminishing virulence or transmissibility [37]. Also, in general, the concept of virulence is multifactorial and comprises several genes and factors. The functional loss of a single gene (*katG*) due to point mutation that is AGC to ACC may lead resistance to INH but need not necessarily compromise virulence. Therefore, the classical concept of less virulent nature of INH^R^ *M. tuberculosis *isolates from south India should be revisited.

Further, the present finding goes in agreement with a study from eastern rural areas of China, which demonstrated that INH^R^ isolates were widely transmitted. Also the correlation of prevalence and transmission between INH^R^ isolates especially with the* katG* S315T substitution and MDR-TB was confirmed. The study also recognized the* katG* S315T mutants among INH^R^ strains, which could be seen as an unsafe factor for subsequent development of MDR-TB. Early detection of the patients with INH^R^ strains would facilitate the modification of treatment regimens and appropriate infection control measures can be taken in time to reduce the risk of further development and transmission of MDR-TB [38]. Additionally, the impact of INH resistance upon treatment outcomes is a matter of great concern. Patients with* katG* S315T substitution are hypothesized to have worse outcomes than those with* inhA* promoter mutations, especially when INH is used in the treatment [39].

Thus, the present study shows a high prevalence of S315T substitution in* katG* gene of INH^R^ clinical isolates of* M. tuberculosis* from south India. Also, this substitution is associated with high level resistance to INH and correlates with multidrug resistance.

## Supplementary Material

The fig-1 shows the amplicon of 209 bp of katG gene in 2-13 lanes from clinical samples and a control at 14th lane. Whereas the Fig. 2 and Fig. 3 represents electropherogram showing the sequences of 209 bp segment of katG gene with the wild type codon (AGC) and the mutant codon (ACC).

## Figures and Tables

**Table 1:** Mutational profile of INH-resistant clinical isolates of *M. tuberculosis *from south India.

Serial number	DST	*katG *	States	Serial number	DST	*katG *	States
1	MDR	ACC	TN	44	MDR	AAC	TN
2	MDR	ACC	TN	45	MDR	ACC	TN
3	MDR	ACC	TN	46	H	AAC	TN
4	H		TN	47	H	AAC	TN
5	H		TN	48	H		TN
6	H		TN	49	H		TN
7	H	ACC	TN	50	H	ACC	TN
8	MDR		TN	51	H	ACC	TN
9	MDR	ACC	TN	52	H	ACC	TN
10	MDR	ATC	TN	53	MDR	ATC	TN
11	MDR	ACC	TN	54	MDR	ACC	TN
12	MDR	ACC	TN	55	H	CGC	TN
13	H		TN	56	MDR		TN
14	H		TN	57	MDR	ACC	TN
15	MDR	ACC	TN	58	H		TN
16	MDR		TN	59	MDR		TN
17	MDR	AAC	TN	60	MDR	ACC	TN
18	H		TN	61	H	ACC	TN
19	H	AAC	TN	62	H	ACC	TN
20	MDR	ACC	TN	63	H		TN
21	MDR	ACC	TN	64	MDR	ACC	TN
22	H		TN	65	MDR	ACC	TN
23	MDR	ACC	TN	66	MDR	ACC	TN
24	MDR		TN	67	MDR	ACC	TN
25	H	ACC	TN	68	MDR	ACC	AP
26	H	ACC	TN	69	H		AP
27	H	ACC	TN	70	H	ACC	AP
28	MDR		TN	71	MDR		AP
29	H		TN	72	MDR		AP
30	MDR		TN	73	MDR	ACC	AP
31	H		TN	74	MDR		AP
32	MDR	AAC	TN	75	MDR		AP
33	H	ACC	TN	76	MDR	ACC	AP
34	H	ACC	TN	77	MDR		AP
35	H	ACC	TN	78	MDR	ACC	AP
36	H	ACC	TN	79	MDR	ACC	AP
37	MDR	ACC	TN	80	H	ACC	AP
38	MDR	ACC	TN	81	H	ACC	AP
39	H	ACC	TN	82	H		KA
40	H	ACC	TN	83	H		KA
41	MDR		TN	84	H	ACC	KA
42	H	ACC	TN	85	H	ACC	K
43	MDR	ACC	TN				

Strains with MIC of 1 mg/liter (number 1–28, 68–85), 5 mg/liter (number 29–45), and >5 mg/liter (number 46–67). Tamil Nadu cultures (number 1–67) and other states cultures (number 68–85). AP: Andhra Pradesh, KA: Karnataka, K: Kerala, H: isoniazid, and MDR: multidrug resistant.
