# Supplementary material for: Investigation of Ser315 Substitutions within katG Gene in Isoniazid-Resistant Clinical Isolates of Mycobacterium tuberculosis from South India
Source: Biomed Res Int. 2015 Jan 28;2015:257983. doi: 10.1155/2015/257983 (PMC4324114; doi:10.1155/2015/257983)
Supplement: Supplementary file 1 — The fig-1 shows the amplicon of 209 bp of katG gene in 2-13 lanes from clinical samples and a control at 14th lane. Whereas the Fig. 2 and Fig. 3 represents electropherogram showing the sequences of 209 bp segment of katG gene with the wild type codon (AGC) and the mutant codon (ACC). [file 257983.f1.doc]

SUPPORTING INFORMATION

**1 2 3 4 5 6 7 8 9 10 11 12 13 14**


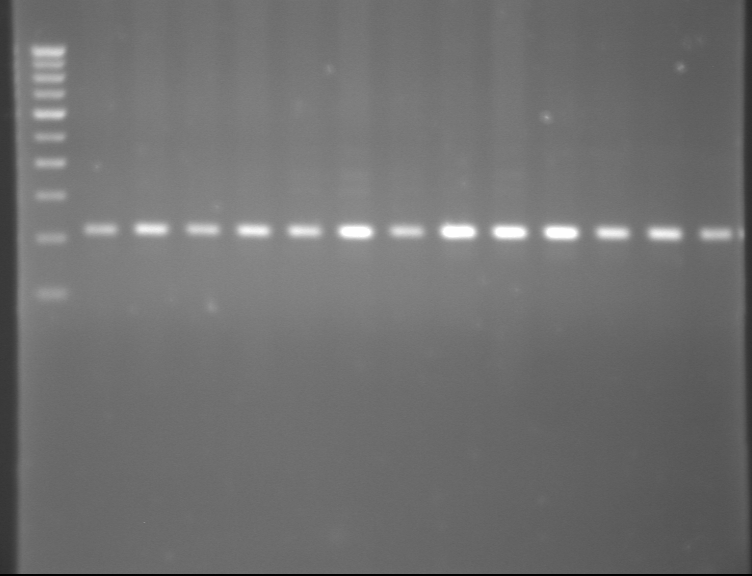


Fig. 1: Amplification of 209bp region of *katG* gene

Lane 1 : 100 bp DNA ladder

Lanes 2-13 : Amplicons

Lane 14 : Positive control-H37Rv


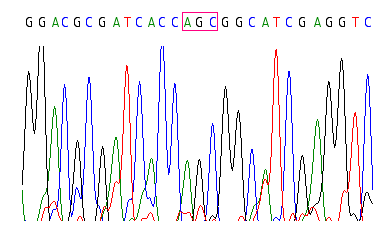


Fig 2. Electropherogram showing the sequences of 209bp segment of *katG* gene with the

Wild type codon (AGC)


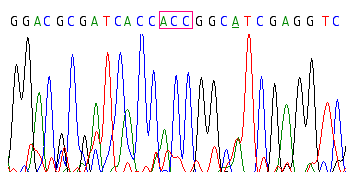


Fig 3. Electropherogram showing the sequences of 209bp segment of *katG* gene with the

Mutant codon (ACC)
